# The critical role of NAT10-mediated N4-acetylcytidine modification in tumor immunity

**DOI:** 10.3389/fimmu.2025.1695495

**Published:** 2025-10-02

**Authors:** Chunhong Li, Xiulin Jiang, YingDong Jia, Qiang Zhou, Yixiao Yuan, Qiang Wang

**Affiliations:** ^1^ Department of Oncology, Suining Central Hospital, Suining, Sichuan, China; ^2^ Department of Systems Biology, City of Hope Comprehensive Cancer Center Biomedical Research Center, Monrovia, CA, United States; ^3^ Department of Gastrointestinal Surgical Unit, Suining Central Hospital, Suining, Sichuan, China

**Keywords:** NAT10, RNA acetylation, N4-acetylcytidine (ac4C), tumor immunity, immune regulation, immune checkpoints, cancer immunotherapy

## Abstract

NAT10, a conserved RNA acetyltransferase, installs N4-acetylcytidine (ac4C) on RNA, thereby regulating stability and translation. Beyond tumor cell proliferation, DNA repair, and chromatin remodeling, NAT10 shapes the tumor immune microenvironment, influencing immune evasion, immune cell infiltration, and responses to immunotherapy. Preclinical studies highlight NAT10 inhibition, such as with Remodelin, as a strategy to enhance cancer treatment-alone or combined with checkpoint blockade, adoptive cell transfer, or chemoradiotherapy. Remaining challenges include *in vivo* validation, greater inhibitor specificity, and biomarker development. This mini-review synthesizes emerging evidence on NAT10 mechanistic roles in tumor immunity and its promise as a therapeutic target.

## 1 Introduction

During tumor initiation and progression, epitranscriptomic and epigenetic modifications are widely recognized as pivotal mechanisms regulating gene expression and immune responses. Among these, RNA modifications (such as m6A, m5C, and ac4C) (1–4), as well as DNA and histone epigenetic modifications, not only contribute to tumor cell proliferation, invasion, and metastasis, but also profoundly shape the tumor immune microenvironment (TIME), thereby modulating antitumor immune responses (5–9).

N-acetyltransferase 10 (NAT10), a highly conserved RNA acetyltransferase, catalyzes the formation of N4-acetylcytidine (ac4C) and regulates RNA stability, splicing, and translational efficiency (10–12). In addition, NAT10 exerts multiple cellular functions, including modulation of histone acetylation, DNA repair, and cell cycle progression, thus playing a critical role in the development and progression of various cancers (13, 14). Recent studies increasingly indicate that NAT10 not only influences the biological behavior of tumor cells, but may also modulate the tumor immune microenvironment by regulating immune-related signaling pathways, cytokine expression, and immune cell functions (4, 15–17). Therefore, a comprehensive investigation of NAT10’s roles and mechanisms in tumor immunity is essential, as it could reveal novel dimensions of immune regulation in cancer and identify potential therapeutic targets for the development of innovative antitumor immunotherapies.

## 2 Structural and biological characteristics of NAT10

**Figure 1:**
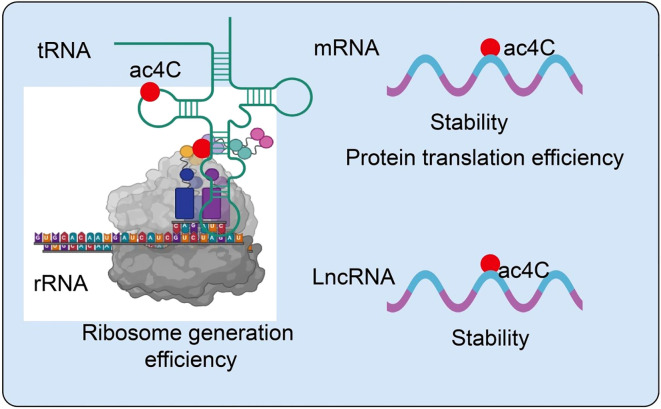
Role of NAT10-mediated RNA acetylation in the regulation of RNA metabolism.

NAT10 is a highly conserved acetyltransferase that introduces N4-acetylcytidine (ac4C) modifications on RNA molecules, thereby modulating RNA stability, translation efficiency, and cellular homeostasis (14). Its enzymatic activity is regulated by multiple signaling pathways, stress responses, and post-translational modifications, allowing context-dependent functions (18). Beyond canonical mRNA and rRNA acetylation, NAT10 can modify noncoding RNAs and chromatin-associated proteins, influencing gene expression and epigenetic regulation (18). Emerging evidence indicates that NAT10-mediated ac4C modifications are critical in maintaining RNA integrity, optimizing translation, and modulating immune responses within the tumor microenvironment (19). These multifaceted roles position NAT10 as a key epitranscriptomic regulator and a potential target for novel cancer therapies. In particular, NAT10-mediated acetylation, especially the formation of ac4C, has emerged as a critical focus in epitranscriptomic research. Accumulating evidence indicates that ac4C modifications are widely distributed across mRNAs, rRNAs, tRNAs, and lncRNAs, playing essential roles in maintaining RNA stability (20, 21), promoting efficient translation, and regulating noncoding RNA functions (22) (**Figure 1**).

## 3 NAT10 and tumor immune microenvironment

The tumor immune microenvironment (TIME) is a complex and dynamic network of immune cells, stromal components, and tumor cells, whose interactions critically influence tumor progression, immune evasion, and therapeutic response. Among the multiple regulators of TIME, NAT10, an RNA acetyltransferase catalyzing N4-acetylcytidine (ac4C) modification, has emerged as a central modulator of tumor immunity across diverse cancer types. NAT10-mediated ac4C modification enhances mRNA stability and translation of key oncogenic and immunoregulatory genes, thereby affecting tumor cell metabolism, cytokine secretion, and immune checkpoint expression. Consequently, NAT10 not only promotes tumor proliferation, angiogenesis, and metastasis but also shapes immunosuppressive landscapes by modulating the abundance, differentiation, and function of tumor-infiltrating regulatory T cells (Tregs), tumor-associated macrophages (TAMs), and effector T cells. Moreover, NAT10 influences the efficacy of immune checkpoint inhibitors (ICIs) and radiotherapy, linking RNA epitranscriptomic modifications to therapeutic sensitivity, while its role in inflammasome activation further connects innate immunity with tumor control. Collectively, these findings position NAT10 as a pivotal orchestrator of tumor-immune interactions, providing a mechanistic basis for its potential as a therapeutic target to remodel the TIME and enhance antitumor immunity.

### 3.1 Tumor-infiltrating regulatory T cells

**Figure 2:**
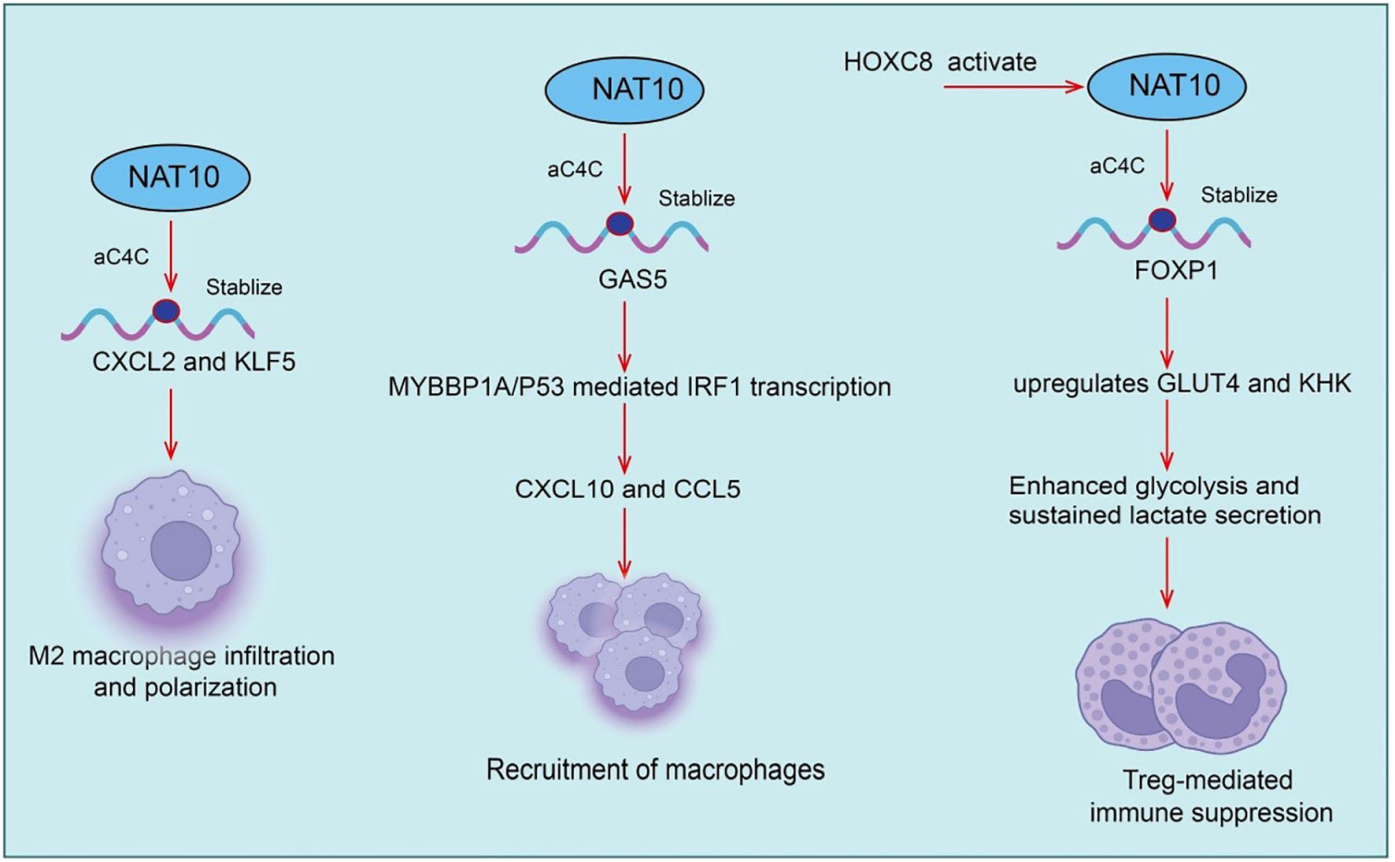
Roles and mechanisms of NAT10-mediated RNA acetylation in tumor-infiltrating regulatory T cells (Tregs) and tumor-associated macrophages (TAMs).

Tumor-infiltrating regulatory T cells (Tregs) are key immunosuppressive populations within the tumor immune microenvironment. They maintain tumor immune tolerance by secreting inhibitory cytokines such as IL-10 and TGF-β, expressing checkpoint molecules including CTLA-4 and PD-1, and suppressing effector T cell activation and proliferation (23–25). Recent studies indicate that NAT10 plays a critical role in modulating Treg infiltration and function. NAT10 catalyzes N4-acetylcytidine (ac4C) modification on mRNAs. This modification promotes glycolysis and the expression of immunosuppressive factors in tumor cells, indirectly enhancing Treg recruitment and suppressive activity within the tumor microenvironment (26). Specifically, NAT10 expression is significantly upregulated in cervical cancer tissues and correlates with poor patient prognosis (27). Mechanistically, HOXC8 binds to the NAT10 promoter, activating NAT10 expression. Increased NAT10 enhances ac4C modification and translation of FOXP1 mRNA. FOXP1 upregulates GLUT4 and KHK, promoting tumor glycolysis. Enhanced glycolysis leads to sustained lactate secretion, which further amplifies Treg-mediated immunosuppression (27). Notably, NAT10 knockdown significantly improves the antitumor efficacy of PD-L1 blockade *in vivo*. Thus, the NAT10/ac4C/FOXP1 axis links tumor glycolytic metabolism to Treg-mediated immune suppression. This highlights NAT10 as both an oncogenic factor in cervical cancer and a potential target for combination therapy with PD-1/PD-L1 blockade (27). Beyond immune suppression, NAT10 also regulates angiogenesis. lncRNA XIST recruits hnRNPK to facilitate YAP1 nuclear translocation, activating TEAD4-dependent VEGFA transcription and promoting angiogenic programming (28). Genetic or pharmacological inhibition of NAT10, for example with Remodelin, reduces VEGFA secretion, enhances pericyte coverage and basement membrane integrity, and normalizes tumor vasculature. In syngeneic gastric cancer models, NAT10 inhibition remodels the immune microenvironment by upregulating CXCL9/10/11 chemokines, increasing cytotoxic lymphocyte infiltration, and reducing Treg proportions (28). Furthermore, combination therapy with Remodelin and the YAP1 inhibitor Verteporfin synergistically enhances anti-PD-1 efficacy, significantly suppressing tumor growth in immunocompetent mouse models (28). Overall, Treg abundance and function are closely linked to NAT10. Targeting NAT10 offers a promising strategy to modulate the tumor immune microenvironment (**Figure 2**).

### 3.2 Tumor-associated macrophages

Tumor-associated macrophages (TAMs) are among the most abundant immune cell populations within the tumor microenvironment (TME) and play critical roles in tumor growth, invasion, angiogenesis, and immunosuppression (29–31). TAMs typically exhibit an M2-like phenotype, secreting pro-tumorigenic factors such as VEGF, TGF-β, and IL-10, thereby suppressing effector T cell function and promoting immune evasion and tumor progression (32–34). Recent studies have shown that in hepatic metastatic gastric cancer (GC) cells, the RNA acetyltransferase N-acetyltransferase 10 (NAT10) is aberrantly overexpressed. Specifically, NAT10-mediated N4-acetylcytidine (ac4C) modification enhances the stability of CXCL2 and KLF5 mRNAs. The secreted CXCL2 subsequently promotes M2 macrophage infiltration and polarization, and through oncostatin M production, activates the STAT3 signaling pathway, transcriptionally upregulating NAT10 expression and forming a positive feedback loop (35). Similarly, in prostate cancer, NAT10 is significantly upregulated, enhancing tumor cell proliferation and invasion. Mechanistically, NAT10 suppresses CD8^+^ T cell recruitment and cytotoxicity via the CCL25/CCR9 axis, thereby establishing an immunosuppressive microenvironment that promotes tumor progression (36). Notably, ac4C modification scores based on NAT10 downstream targets can predict immune infiltration levels and response to immunotherapy, providing a potential tool for patient stratification(**Figure 2**).

In addition, long non-coding RNA GAS5 has been shown to exert tumor-suppressive effects within the TME. Clinical data indicate a positive correlation between GAS5 levels and macrophage and T cell infiltration (37). *In vitro* and *in vivo* experiments demonstrate that GAS5 overexpression promotes recruitment of both macrophages and T cells. Mechanistically, GAS5 directly binds MYBBP1A to stabilize p53 and enhance MYBBP1A–p53 interaction, thereby promoting p53-mediated IRF1 transcription, activating the type I interferon signaling pathway, and increasing downstream chemokines CXCL10 and CCL5, ultimately enhancing immune cell infiltration (37). Activation of type I interferon signaling has been closely associated with improved efficacy of immune checkpoint blockade in non-small cell lung cancer (NSCLC). Collectively, TAM abundance, phenotype, and function are closely linked to NAT10/ac4C modification, while tumor-derived GAS5 enhances immune cell infiltration via the MYBBP1A–p53/IRF1 axis, potentially improving immunotherapy responses (37). These findings highlight targeting NAT10 and its regulatory network as a promising strategy to remodel the immune microenvironment and enhance antitumor immunity.

### 3.3 T cells differentiation and effector

**Figure 3:**
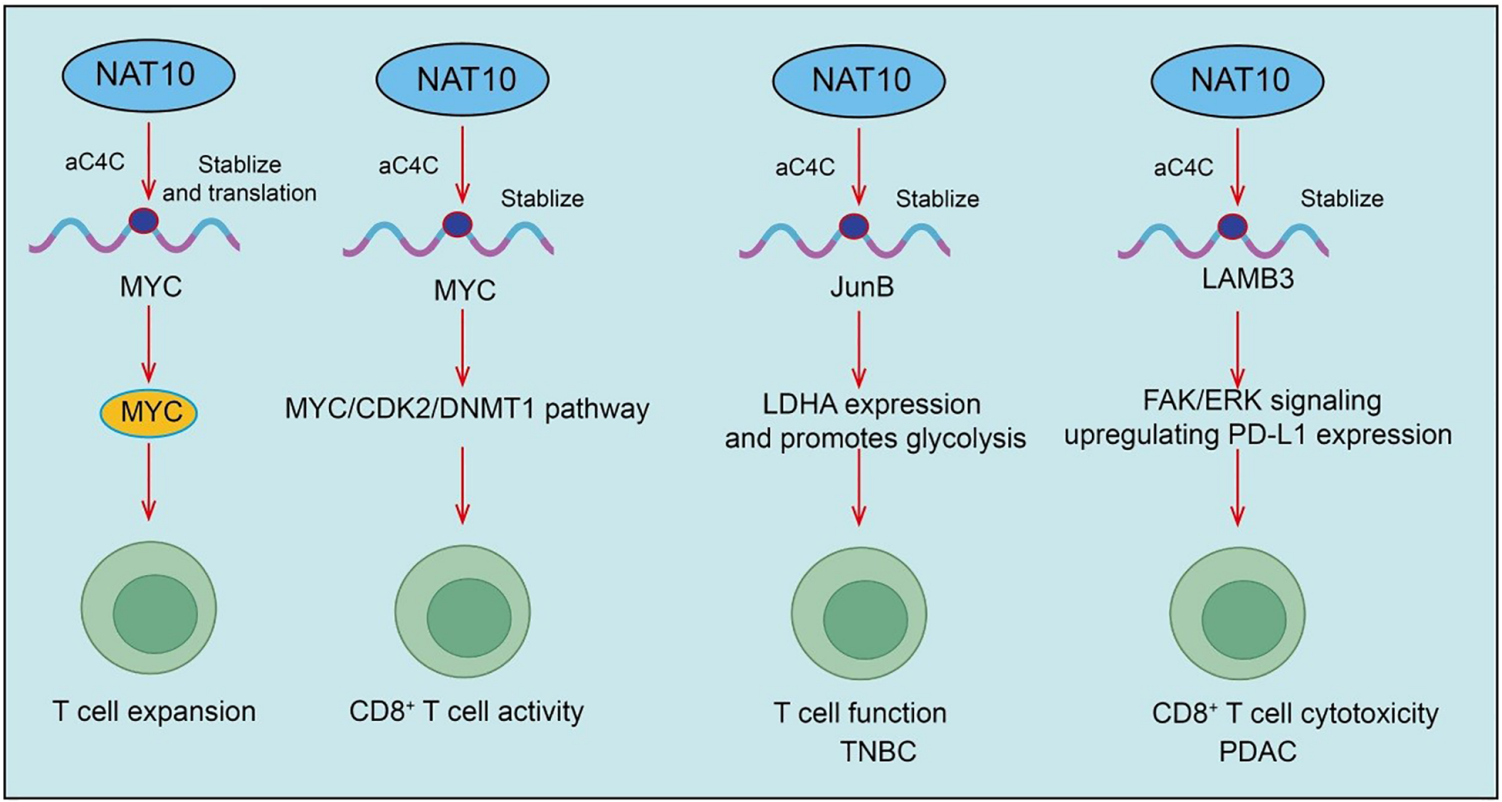
Roles and mechanisms of NAT10-mediated RNA acetylation in T cell activation and effector functions.

T cells are central effector cells in antitumor immunity, mediating differentiation, proliferation, cytotoxicity, and immune memory formation (38). Naive T cells, upon antigen stimulation, differentiate into diverse subsets including effector CD8^+^ T cells, helper CD4^+^ T cells, and memory T cells (39). However, within the tumor microenvironment, chronic antigen stimulation, metabolic stress, and immunosuppressive signals often lead to T cell exhaustion, characterized by reduced proliferative capacity, diminished cytotoxicity, and upregulation of inhibitory checkpoint molecules such as PD-1, TIM-3, and LAG-3 (40). Recent evidence indicates that NAT10 is upregulated in multiple tumor types and negatively correlates with immune cell infiltration and overall patient survival (4). Specifically, NAT10 deficiency in tumor cells enhances tumor-specific cellular immune responses, thereby inhibiting tumor growth. Mechanistically, MYC is a key downstream target of NAT10 and is regulated via ac4C modification of its mRNA. NAT10 inhibition blocks the MYC/CDK2/DNMT1 pathway, promotes double-stranded RNA formation, and activates type I interferon responses, thereby enhancing tumor-specific CD8^+^ T cell activity *in vivo (* 41). Importantly, pharmacological inhibition of NAT10 using small molecules (e.g., Remodelin) or PEI/PC7A/siNAT10 nanoparticles synergizes with PD-1 blockade, boosting antitumor immunity and restraining tumor progression(**Figure 3**).

A recent landmark study revealed that NAT10, the enzyme mediating ac4C modification, plays a crucial role in the progression of nasopharyngeal carcinoma (NPC). Specifically, the authors found that high NAT10 expression significantly promotes NPC progression and correlates with poor patient prognosis. NAT10 enhances the stability and translation efficiency of CEBPG, DDX5, and HLTF mRNAs through ac4C modification, with the NAT10/ac4C/DDX5 axis upregulating HMGB1 expression and suppressing CD4+ and CD8+ T cell activity (42). Inhibition of NAT10 increases sensitivity to PD-1 therapy (42). In summary, this study reveals the critical role of NAT10-mediated ac4C modification in NPC immune evasion and therapeutic response, providing a potential strategy for NAT10-targeted immunotherapy (42). In triple-negative breast cancer (TNBC), NAT10 loss similarly suppresses tumor development and promotes T cell activation. Mechanistic studies reveal that NAT10 enhances JunB mRNA ac4C modification, upregulating JunB expression, which subsequently increases LDHA expression and promotes glycolysis (43). Furthermore, Remodelin treatment elevates T cell surface CTLA-4 expression, and combination therapy with anti-CTLA-4 antibody further activates T cells and suppresses tumor progression (43). In pancreatic ductal adenocarcinoma (PDAC), NAT10 is upregulated in both tissues and cell lines, correlating with poor progression-free survival (PFS). Within the PDAC tumor microenvironment, NAT10 promotes subcutaneous tumor growth, increases exhausted CD8^+^ T cells (CD8^+^Tex), particularly the intermediate CD8^+^Tex subset, while reducing cytotoxic CD8^+^ T cells (CD8^+^Tc) (44). Treatment with Naamidine J significantly restores CD8^+^Tc proportions and reduces intermediate CD8^+^Tex, thereby improving antitumor immunity. Mechanistically, NAT10 stabilizes LAMB3 mRNA through ac4C modification, activating the FAK/ERK signaling pathway, upregulating PD-L1 expression, and suppressing CD8^+^ T cell cytotoxicity (44). PD-1/PD-L1 blockade can restore CD8^+^ T cell killing capacity, ultimately impacting tumor progression (**Figure 3**).

Moreover, during T cell activation, naive resting T cells must exit quiescence and rely on global protein translation for rapid expansion (45, 46). NAT10 expression is upregulated upon activation and mediates ac4C modification of Myc mRNA. ac4C-modified Myc mRNA exhibits enhanced translational efficiency, enabling rapid MYC protein synthesis and supporting T cell expansion (47). Conditional Nat10 deletion in mouse T cells causes severe cell cycle arrest and impaired proliferation due to MYC deficiency, exacerbating infection in acute lymphocytic choriomeningitis virus models (47). Additionally, T cells from elderly individuals with reduced NAT10 levels display proliferative defects, which may partially explain diminished antiviral immunity with aging. Collectively, T cell differentiation, exhaustion, and effector function are closely associated with NAT10 and ac4C modification, revealing novel regulatory mechanisms within the tumor immune microenvironment and suggesting potential strategies to enhance immunotherapy by targeting NAT10 (47).

### 3.4 Efficacy of immune checkpoint inhibitors

**Figure 4:**
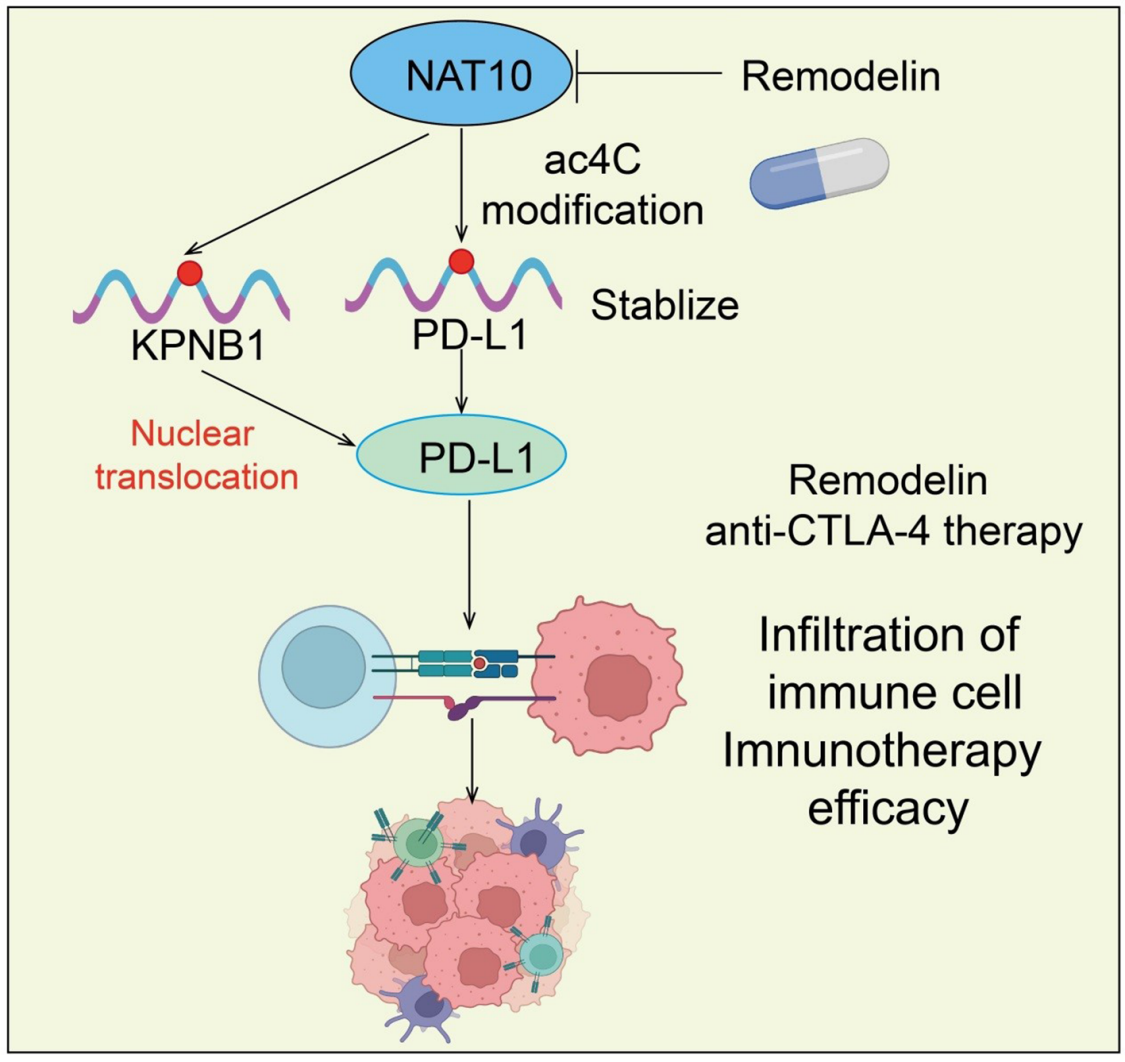
Roles and mechanisms of NAT10-mediated RNA acetylation in modulating the efficacy of immune checkpoint inhibitors.

Immune checkpoint inhibitors (ICIs), including antibodies targeting PD-1/PD-L1 and CTLA-4, restore antitumor immunity by releasing T cells from immunosuppressive constraints within the TME (48, 49). However, ICI efficacy is influenced by multiple factors, including the status of the tumor immune microenvironment, T cell infiltration, metabolic reprogramming, and expression of immunosuppressive factors. As a key checkpoint, PD-1/PD-L1 plays a pivotal role in various cancers (50). Studies have shown that NPM1 enhances PD-L1 transcription via acetylation, while NAT10 promotes NPM1 acetylation, further upregulating PD-L1 expression. Importantly, the NAT10 inhibitor Remodelin effectively reduces NPM1 acetylation and subsequently decreases PD-L1 expression (51). *In vivo* experiments demonstrate that Remodelin combined with anti-CTLA-4 therapy more effectively suppresses tumor growth than either treatment alone, suggesting that NAT10-mediated regulation of PD-L1 may represent a novel approach to optimize ICI therapy (51, 52) (**Figure 4**).

Furthermore, radiotherapy (RT) resistance is a major cause of tumor recurrence in NSCLC. RT-resistant cells (PC9R and A549R) exhibit elevated ac4C and NAT10 levels. NAT10 knockdown not only inhibits cell proliferation but also enhances immune function by upregulating TNF-α and IFN-γ while downregulating PD-1 and TIM-3 (53). Mechanistically, NSCLC RT resistance is mediated by NAT10-dependent ac4C modification of KPNB1, which facilitates PD-L1 nuclear translocation and enhances immune evasion in resistant cells (53). Thus, NAT10 promotes RT resistance in NSCLC via ac4C-mediated upregulation of KPNB1 and PD-L1 nuclear translocation, providing new insights into RT resistance mechanisms and potential intervention strategies (**Figure 4**). Additionally, kynurenine suppresses T cell activation and promotes tumor immune evasion while enhancing NAT10-mediated ac4C modification. NAT10 inhibition strengthens T cell activation and inhibits immune escape. Mechanistic studies reveal that NAT10-mediated ac4C modification of PD-L1 mRNA promotes immune escape by enhancing PD-L1 expression, which suppresses T-cell activation (54). Collectively, NAT10 serves as a central regulator of tumor immunity, with its expression and activity directly affecting ICI efficacy, highlighting a potential target to improve immunotherapy outcomes.

### 3.5 NAT10 and inflammasome regulation

The inflammasome is a key multiprotein complex in innate immunity, responsible for sensing pathogens and cellular stress, leading to caspase-1 activation and secretion of pro-inflammatory cytokines IL-1β and IL-18. Recent studies suggest that NAT10, through its N4-acetylcytidine (ac4C) modification, can influence inflammasome activation by stabilizing critical viral and host RNAs. This RNA epitranscriptomic regulation links NAT10 activity to innate immune responses and highlights its potential as a target for modulating the immune microenvironment and developing novel antitumor immunotherapies (55, 56). This process is critical for antimicrobial defense and antitumor immunity. Recent studies indicate that NAT10 and its catalyzed N4-acetylcytidine (ac4C) modification not only stabilize HIV-1 transcripts but also play key roles during Kaposi’s sarcoma-associated herpesvirus (KSHV) infection (57). Specifically, NAT10 catalyzes ac4C modification on KSHV-encoded polyadenylated nuclear RNA (PAN RNA), a long non-coding RNA, triggering viral transition from latency to lytic replication. Mutation of ac4C sites on PAN RNA abolishes its modification, reducing lytic gene expression and viral particle production (57). Similarly, NAT10 knockdown or mutation destabilizes PAN RNA, preventing KSHV activation. Notably, PAN RNA ac4C modification also facilitates NAT10 recruitment to IFI16 mRNA, promoting its ac4C modification, enhancing mRNA stability and translation efficiency, and ultimately activating the inflammasome (57). In summary, NAT10 regulates inflammasome activation via ac4C modification, linking RNA epitranscriptomic regulation to innate immune responses and providing a potential target for modulating the immune microenvironment and developing antitumor immunotherapies.

## 4 Therapeutic implications of targeting NAT10

NAT10, as the “writer” of ac4C modifications, regulates mRNA stability and translation, DNA damage repair, chromatin state, and cell cycle progression (58). Consequently, NAT10 inhibitors have emerged as promising anticancer agents. Remodelin and its derivatives are the most extensively studied small-molecule inhibitors (59). Remodelin effectively inhibits NAT10 acetyltransferase activity, corrects nuclear structural abnormalities, and diminishes tumor cell survival under DNA damage stress. In multiple cancer models, Remodelin sensitizes tumor cells to radiotherapy and chemotherapy, enhancing DNA damage-induced cell death (60).

Despite these promising effects, NAT10 inhibitor development faces pharmacological challenges, including limited selectivity, poor oral bioavailability, and suboptimal *in vivo* stability (61). Future strategies aim to improve target specificity, develop covalent or allosteric inhibitors, optimize pharmacokinetics, and achieve tissue-specific delivery via prodrugs or nanocarriers (62). These improvements are intended to maximize antitumor efficacy while minimizing toxicity to high-turnover tissues such as hematopoietic and mucosal compartments.

Beyond direct cytotoxicity, NAT10 inhibition significantly impacts the tumor immune microenvironment (TIME). By altering tumor cell immunogenicity, antigen processing and presentation, interferon signaling, and immunosuppressive factor secretion, NAT10 modulates immune cell infiltration and activity (63). Specifically, NAT10 inhibition reduces immunosuppressive chemokines and cytokines, enhances antigen presentation, and amplifies DNA damage–induced cGAS–STING–type I interferon signaling, thereby improving the efficacy of immune checkpoint inhibitors (PD-1/PD-L1 or CTLA-4) (64). NAT10 inhibitors can also synergize with radiotherapy or chemotherapy to increase immunogenic cell death (ICD) and neoantigen release. This creates a potential “triple therapy” strategy with ICIs, converting immunologically “cold” tumors into “hot” tumors and enhancing immunotherapy responsiveness (65). In adoptive cell therapies, NAT10 inhibition may relieve immunosuppression in the TIME and increase tumor susceptibility to effector cells such as CAR-T, CAR-NK, or TILs, by upregulating apoptotic pathways and reducing inhibitory ligand expression (66). Additionally, precise modulation of NAT10/ac4C within effector cells may optimize metabolic fitness and translational efficiency, enhancing persistence and cytotoxicity, though safety considerations remain. NAT10 inhibitors may also synergize with innate immune activators (e.g., STING or TLR agonists, oncolytic viruses) to further boost type I interferon signaling and antitumor immunity (67). Overall, targeting NAT10 not only exerts direct tumor-suppressive effects but also reshapes the TIME to enhance immunotherapeutic efficacy. Future efforts should prioritize the development of potent and safe NAT10 inhibitors, explore rational combination therapies with immunomodulatory approaches, and identify biomarkers to guide individualized therapy. This strategy holds promise for translating NAT10-targeted interventions into clinical applications.

## 5 Challenges and future perspectives

Despite significant progress in understanding NAT10 and its ac4C-mediated RNA acetylation, several challenges remain. First, most studies are limited to *in vitro* cellular models or mechanistic analyses. There is a lack of systematic animal models and clinical sample validation, leaving NAT10’s precise role in tumor immune regulation incompletely defined (68). Second, NAT10 has multiple substrates and diverse biological functions, including RNA modification, histone acetylation, DNA repair, and cytoskeletal regulation. Therefore, global NAT10 inhibition may cause significant toxicity, posing challenges for drug development (69). Third, currently available NAT10 inhibitors, such as Remodelin, remain chemical probes with limited selectivity and suboptimal pharmacokinetics, far from clinical translation.

Future research should address several key questions. First, which biomarkers can stratify patients likely to respond to NAT10-targeted therapy? Candidate biomarkers may include NAT10 expression levels, ac4C modification signatures, FOXP1 activity, glycolytic or lactate-associated markers, and immune cell infiltration profiles. Second, how does NAT10 inhibition reshape the tumor immune microenvironment (TIME) *in vivo*? Studies using transgenic animal models and patient-derived clinical samples are essential to bridge preclinical findings to clinical applications (70). Third, can novel NAT10 inhibitors be developed with higher specificity, improved pharmacokinetics, and minimized toxicity? Approaches may include structure-guided allosteric inhibitors, reversible or covalent binders, and precision-targeted agents using nanodelivery or prodrug strategies. Additionally, understanding resistance mechanisms to NAT10 inhibition will inform rational combination therapies. Integrating NAT10 inhibition with immunotherapy, radiochemotherapy, or targeted therapies could enhance antitumor efficacy, convert “cold” tumors into “hot” tumors, and improve patient outcomes. Finally, establishing reliable biomarkers for patient stratification, efficacy prediction, and monitoring immune modulation will support personalized NAT10-targeted therapy. As mechanistic insights deepen and drug development progresses, NAT10 is poised to become a pivotal target in tumor immunotherapy, offering new therapeutic strategies and clinical opportunities.

## 6 Conclusion

In recent years, the role of NAT10, an RNA acetyltransferase, in tumor immunity has gradually been elucidated. By catalyzing ac4C modifications, NAT10 exerts critical regulatory functions in mRNA stability, translational efficiency, and the activity of lncRNAs and tRNAs, thereby influencing the expression of numerous immune-related genes and the activity of key signaling pathways. Accumulating evidence indicates that NAT10 not only plays a pivotal role in tumor cell proliferation and survival but also shapes antitumor immune responses by modulating T cell function, myeloid cell differentiation, and the expression of immunosuppressive factors. These findings position NAT10 as an emerging nexus connecting the epitranscriptome with tumor immunology.

Looking ahead, NAT10 holds promise as a potential novel target for cancer immunotherapy. On one hand, targeting NAT10 may directly suppress malignant tumor cell properties; on the other hand, its regulatory role in the immune microenvironment provides a mechanistic rationale for combination strategies with immune checkpoint inhibitors, cellular therapies, and radiochemotherapy. With the ongoing development of next-generation NAT10 inhibitors and the establishment of associated biomarkers, NAT10 has the potential to advance precision medicine and individualized immunotherapeutic approaches. Although current research remains at an early stage and requires further *in vivo* and clinical validation, NAT10 represents a highly promising therapeutic target that may have broad implications in future cancer immunology and treatment.
